# The Influence of Family Caregivers’ Experience of Interprofessional Care on Their Participation in Health Checkups as Preventive Health Behavior in Japan—A Cross-Sectional Analysis

**DOI:** 10.3390/ijerph18010223

**Published:** 2020-12-30

**Authors:** Gen Nakayama, Shoichi Masumoto, Junji Haruta, Tetsuhiro Maeno

**Affiliations:** 1 Department of Primary Care and Medical Education, Graduate School of Comprehensive Human Sciences, University of Tsukuba, 1-1-1, Tennodai, Tsukuba, Ibaraki 305-8576, Japan; s1830420@s.tsukuba.ac.jp; 2 Department of Family Medicine, General Practice and Community Health, Faculty of Medicine, University of Tsukuba, 1-1-1, Tennodai, Tsukuba, Ibaraki 305-8576, Japan; 3 Medical Education Center, School of Medicine, Keio University, 35, Shinano-machi, Shinjuku-ku, Tokyo 160-8582, Japan; junharujp@keio.jp; 4 Department of Primary Care and Medical Education, Faculty of Medicine, University of Tsukuba, 1-1-1, Tennodai, Tsukuba, Ibaraki 305-8576, Japan; maenote@md.tsukuba.ac.jp

**Keywords:** caregivers, health behavior, health care quality assurance, health services evaluation, integrated care

## Abstract

Background: The role of family caregivers has been vital, especially in superaging societies like Japan’s. The caregivers’ experience of interprofessional care is a key aspect in their evaluation of the quality of integrated care. We sought to explore whether family caregivers’ experience of interprofessional care is associated with their own participation in health checkups as preventive health behaviors. Methods: We used cross-sectional data obtained during the development of the Japanese version of the Caregivers’ Experience Instrument (J-IEXPAC CAREGIVERS). Participants who had provided care for at least one year were surveyed (*n* = 251). We assessed family caregivers’ experience of interprofessional care using J-IEXPAC CAREGIVERS and their participation in health checkups. Results: Multivariate logistic regression analysis revealed that the J-IEXPAC CAREGIVERS total score was significantly associated with the caregivers’ participation in health checkups [odds ratio per 1-point increase = 1.05; 95% confidence interval 1.01–1.09]. Two domain scores (attention for the patient and attention for the caregiver) of J-IEXPAC CAREGIVERS were significantly associated with the outcome. Conclusions: Family caregivers with more positive experiences of interprofessional care were more likely to participate in health checkups. These results support the significance of family caregivers’ experience of care, which may promote preventive health behaviors.

## 1. Introduction

With a high proportion of the world population experiencing aging and an increasing incidence of chronic diseases, the role of the family caregiver has become critical. The impact of caregiving responsibilities on family caregivers’ physical, psychological, and social well-being has been noted [1,2,3]. Family caregivers have been shown to neglect their own health and self-care and to be less likely to engage in preventive health measures [1,2]. The need to encourage family caregivers to take care of their own health and advise on health-promoting behaviors has been identified, as maintaining the health of the caregiver has been demonstrated to be a critical factor in enabling them to continue their provision of care [4].

When focusing on preventive health behaviors as a component of the health of family caregivers, periodic health checkups are a recommended preventive health behavior for family caregivers. Research experts in the fields of family caregivers’ health and self-care have reported that there should be annual health checks for every caregiver [5]. The Centers for Disease Control and Prevention’s web page on family caregivers claims that “encourage caregivers to get regular check-ups [6].” Although there is no standard definition of health checkups (health checks or health examinations), the Cochrane Review in 2019 defined general health checks as screening for >1 disease or risk factor and in >1 organ system and excluded screening for single diseases [7]. As this review covers trials that include cancer screening, health checkups often involve cancer screening. While the review showed that general health checks in adults aged 20 to 64 y unselected for disease or risk factors are unlikely to reduce morbidity and mortality, it is unclear whether the results can be applied to family caregivers. Family caregivers with more than a certain number of hours of providing care have been shown to have a higher risk of cardiovascular disease than non-caregivers [8,9]. The benefits of health checkups among family caregivers, who are generally considered to be at risk of physically and emotionally burden [1,2], may be more meaningful than those of the general population.

In Japan, where the government recommends several types of annual health checkups depending on the subject [10], health checkups among family caregivers have also attracted attention as their preventive health behaviors. An example of an adult health checkup is an annual specific health checkup targeted at ages 40–74 y to prevent lifestyle-related diseases, which consist of a medical consultation and examinations of items such as blood pressure, urine, and blood [10]. Any type of health checkup includes at least screening for chronic diseases such as hypertension and diabetes. Depending on the type of health checkups, the checkups may also include cancer screening (e.g., fecal occult blood testing for colorectal cancer screening). Previous observational studies in Japan have shown the effectiveness of health checkups [11,12]. Focusing on family caregivers, a study suggests that while female family caregivers are at higher risk of hypertension compared with non-caregivers, the caregivers have had fewer annual health checkups and therefore need some support to receive these [13]. It was also reported that a higher care-need level in care recipients was negatively associated with family caregivers’ participation in health checkups [14].

A variety of individual-level and interpersonal factors influence preventive health behaviors. One of these is social support. Social support refers to the process by which interpersonal relationships promote and protect individual well-being, especially in the face of stressful living conditions [15]. Previous studies have shown that social support is positively associated with cancer screening participation [16,17,18]. Theoretical models indicate that social support prevents and buffers stress; increases connectedness, control and self-esteem; and consequently promotes healthy behaviors, including preventive health practices [19]. For instance, one study revealed that social support is associated with repeated breast cancer screening in low-income female caregivers [16].

Although these studies have mainly focused on social support gained from personal *informal* relationships, *formal* social support has also attracted attention when considering help for family caregivers [20,21]. While informal social support is provided by family members, neighbors and friends, formal social support is provided by organizations, health care and social care professionals who support patients and their family caregivers.

Efforts to assess the quality of formal support provided by such professionals have included the experience of family caregivers. Family caregiver experience has joined patient experience as a key aspect in evaluating quality of care [22]. When evaluating care for the frail and elderly and their family caregivers, the caregivers are thought to be in the best position to assess such care [23]. We previously developed a Japanese version of a scale to measure family caregiver experience of interprofessional care for patients and families [24]. This instrument was originally developed in Spain to measure the quality of integrated health and social care [25], and includes elements of professional attention to the health and well-being of family caregivers themselves. This scale can be used to assess the quality of health and social care from the caregivers’ perspective.

Given the association of social support with preventive health behavior, the quality of formal support from family caregivers’ perspective (i.e., family caregivers’ experience of interprofessional care) may also be related to their preventive health behavior. To our knowledge; however, the relationship between these variables has not been clarified. Researchers of patient experience are increasingly interested in understanding how patient experience is associated with other measures of clinical processes or outcomes. Such knowledge could help providers improve the efficiency and effectiveness of care [26]. Similarly, examining the relationship between family caregivers’ experience and their preventive health behavior may emphasize to providers the importance of reflecting on their quality of care through the family caregivers’ experience.

In this study, we aimed to explore whether family caregivers’ experience of interprofessional care is associated with their participation in health checkups. We used cross-sectional data obtained during the development of the Japanese version of the scale.

## 2. Materials and Methods

### 2.1. Participants and Procedures

The cross-sectional data for this study were drawn from the development study on the Japanese version of the scale [24]. As reported in that study, recruitment for participation occurred between October and November 2019 and involved questionnaires administered for 400 family caregivers, who were caring for their community-dwelling patients under the supervision of care managers. Care managers support family caregivers and their patients by managing and coordinating the roles of other healthcare and social care professionals under Japan’s long-term care insurance system.

Study participants in the development study on the Japanese version of the scale were eligible if they were “family caregivers (not receiving economic remuneration for providing care and considered related to the patient),” aged ≥20 y, caring for patients who were suffering from “chronic conditions” [27], caring for ≥6 months, and able to read the Japanese questionnaire. “Family” was defined as “a group of individuals with a continuing legal, genetic and/or emotional relationship” [28]. A researcher instructed the care managers to request that the primary caregiver complete the questionnaire whenever possible when a patient had multiple caregivers. The participants provided informed consent via the questionnaires.

**Figure 1:**
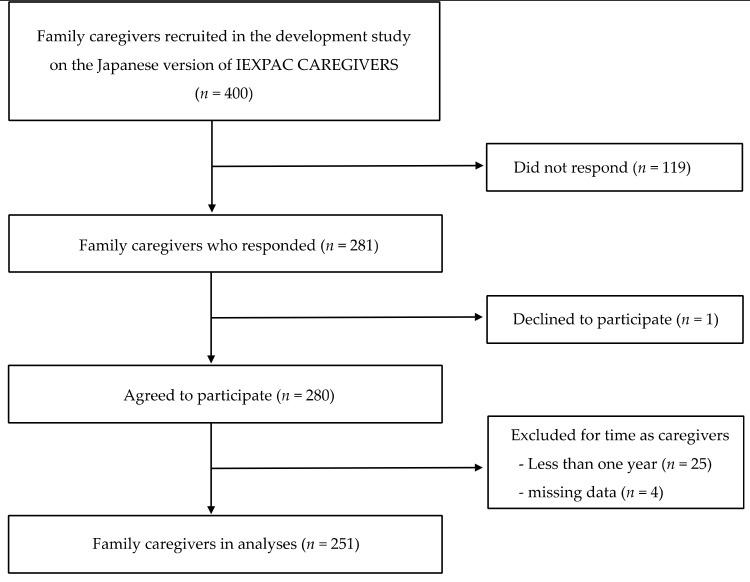
Participants flow chart.

Of the 400 family caregivers recruited, 251 were included in the present study (Figure 1). Because the outcome was to ask whether family caregivers had participated in health checkups within the past year, as described below, the study excluded family caregivers who had provided care for <1 y and those whose length of care was missing (Figure 1).

### 2.2. Outcome Variable

The main outcome variable was whether family caregivers had undergone any health checkups in the past year. Referring to the Comprehensive Survey of Living Conditions questionnaire administered by the government [29], the number of caregivers undergoing health checkups was derived from the following question: “Have you had any health checkups (a health checkup or a thorough medical checkup) in the past year?” It excluded dental checkups or examinations as a medical practice in a hospital or clinic. In Japan, annual health checkups are mainly conducted by municipalities, employers, or insurers, and are distinct from doctor visits covered by the Japanese health insurance system. In the Comprehensive Survey of Living Conditions questionnaire, screening for cancer only is excluded from health checkups, and the questions about cancer screening are in a separate part of the questionnaire. However, because the volume of our questionnaire was limited, our questionnaire did not exclude screening for cancer only from questions about health checkups.

### 2.3. Family Caregivers’ Experience of Interprofessional Care

We used the Japanese version of the Caregivers’ Experience Instrument (J-IEXPAC CAREGIVERS). The Caregivers’ Experience Instrument, also called IEXPAC CAREGIVERS, is a scale which measures family caregivers’ experience of integrated health and social care for both patients with chronic conditions and their family caregivers [25]. This scale was developed and validated in Spain, based on the Instrument to Evaluate the EXperience of PAtients with Chronic Diseases (acronym in Spanish: IEXPAC), which is theoretically based on the Chronic Care Model and is inspired by patient-centered integrated care approaches [30].

J-IEXPAC CAREGIVERS is a self-reported questionnaire with 16 items: 12 items and four additional questions related to situation. This scale consists of two dimensions—attention for the patient and attention for the caregiver—according to factor analysis. For example, the former dimension consists of *They respect the lifestyle of the person I care for* and the latter of *They are concerned about my health and wellbeing*. Each item is rated on a five-point Likert scale range from 1 (Never) to 5 (Always). As items are not weighted, a scale score is calculated by simply summing the scores for each of the 12 items, but not the additional questions; the possible total score lies in the range 12–60. A high total score indicates a high quality of interprofessional care from the caregiver’s perspective. We previously confirmed the validity and reliability of J-IEXPAC CAREGIVERS [24].

### 2.4. Other Variables

We identified other variables that may confound the association between family caregivers’ experience of interprofessional care and their participation in health checkups based on a literature review. We included variables for age, gender, educational attainment, household equivalized income, and burden on the family caregivers, as well as care-need level of the patients. Household equivalized income was calculated as the gross income divided by square root of the number of household members; the gross income was measured in classes (e.g., 1–1.99 million JP¥) and mid-point of the class (e.g., 1.5 million JP¥) was used [31]. The equivalent income was categorized into quartiles. Caregiver burden was assessed using the short version of the Japanese version of the Zarit Caregiver Burden Interview which total score ranges from 0 to 32 (higher score means higher burden) [32]. The caregiver burden scale suggests that caregivers are at risk of depression if they score 13 or more [33]. The care-need level was classified as “support required” (2 levels) or “care required” (5 levels) according to Japan’s long-term care insurance system [34]. Based on a previous study, we apportioned the care-need level into three categories: lower need level (support required levels 1 and 2), middle need level (care required levels 1–3) and higher need level (care required levels 4 and 5) [14]. We defined gender, educational attainment, household equivalized income, and care-need level as categorical variables.

### 2.5. Statistical Analysis

Descriptive statistics were obtained for participants’ characteristics. Unadjusted associations between participants’ characteristics and the outcome measure were analyzed using the chi-squared test for categorical variables and the Mann–Whitney test for continuous variables. An unadjusted association between the total score for J-IEXPAC CAREGIVERS and the outcome was analyzed using the independent samples *t*-test. In these analyses, missing values were treated by pairwise exclusion.

We used multivariate logistic regression analysis to determine whether the total score for J-IEXPAC CAREGIVERS was positively associated with participation in health checkups. The following possible confounders were included in the analysis: age, gender, educational attainment, household equivalized income and burden of the family caregivers; care-need level of the patients. To examine the multi-collinearity of the independent variables, we analyzed Spearman’s rank correlation coefficient and checked variance inflation factors using multiple regression analysis. In addition, we also performed exploratory analyses of participation in health checkups in relation to J-IEXPAC CAREGIVERS domain scores (attention for the patient and attention for the caregiver) using the same model as the primary analysis. In the exploratory analyses, we repeated the comparisons without the Bonferroni correction.

In the verification of the sample size in the logistic regression analysis, events per variable values of ≥10 were necessary [35]. In this study, with seven independent variables, about 250 samples were needed. Therefore, in the logistic regression analysis, we accounted for missing data for independent variables by using multiple imputation with a fully conditional specification. We used the method at each item level (not at the scale level) for the score of J-IEXPAC CAREGIVERS. Statistical analyses were conducted using SPSS Statistics version 26 (IBM Corp, Armonk, NY, USA).

### 2.6. Ethical Considerations

The study was approved by the Ethics Committee of the Faculty of Medicine, University of Tsukuba (No. 1417-1). All participants were volunteers and checked the box on the questionnaire indicating their intention to participate.

## 3. Results

Table 1 shows the distribution of the 251 participants’ characteristics. The majority of family caregivers were women (77.7%), aged ≥58 y (75.3%), with less than a college education (58.6%) and scored <13 points on the short version of the Japanese version of the Zarit Caregiver Burden Interview (61.9%). The majority of patients had middle care-need level (care required level 1–3) (61.8%). Although not shown in the table, the following primary health conditions were the most common among patients: neurological disorders including dementia (25.9%), stroke (21.9%) and joints/spinal cord/bone fracture (17.1%). The overall proportion of family caregivers who had participated in health checkups in the past year was 72.5%.

Table 2 shows the mean and standard deviation of the J-IEXPAC CAREGIVERS scores overall and by caregiver participation in health checkups. The average J-IEXPAC CAREGIVERS total score was 40.5 out of 60 points. The J-IEXPAC CAREGIVERS total score was significantly associated with family caregivers’ participation in health checkups. The univariate associations between the J-IEXPAC CAREGIVERS domain scores and the outcome were also significant.

Table 3 shows the results of multivariate logistic regression analysis investigating the associations of the J-IEXPAC CAREGIVERS total score with family caregivers’ participation in health checkups. After adjustment for possible confounders, the J-IEXPAC CAREGIVERS total score was significantly associated with family caregivers’ participation in health checkups (odds ratio (OR) per 1-point increase = 1.05; 95% confidence interval (CI) 1.01–1.09). The coefficient of correlation among all the independent variables was <0.5 and all variance inflation factors were ≤1.3, indicating there was no problem with multi-collinearity.

Table 4 shows the results of multivariate logistic regression analysis investigating the associations of the J-IEXPAC CAREGIVERS domain scores with family caregivers’ participation in health checkups. The domain scores were also positively associated with family caregivers’ participation in health checkups: attention for the patient (OR per 1-point increase = 1.08; 95% CI 1.01–1.15), and attention for the caregiver (OR per 1-point increase = 1.12; 95% CI 1.02–1.24).

## 4. Discussion

Our results showed that family caregivers’ experience of interprofessional care for patients and the caregivers was positively associated with the caregivers’ participation in health checkups. This association persisted after adjustment for possible confounders. In addition, two domains of J-IEXPAC CAREGIVERS, which reflects attention for the patient and attention for the caregiver, were associated with the caregivers’ participation in health checkups. Our study indicated the significance of family caregivers’ experience of interprofessional care in the context of their preventive health behaviors.

Considering that family caregivers’ experience of interprofessional care reflects one aspect of the quality of social support, especially formal support provided by healthcare and social care professionals, the results of this study are consistent with those of previous studies that have examined the association between social support and disease screening participation [16,17,18] or health behavior in family caregivers [36]. Messina and Lane et al. showed that emotional/informational support, a component of social support, is associated with repeated breast cancer screening in low-income female caregivers [16]. They measured emotional/informational support as reflecting the availability of someone to share expressions of positive affect, offer empathetic understanding, encourage one to express feelings, and offer advice, information, guidance, or feedback. The key components of J-IEXPAC CAREGIVERS include professional support to improve family caregivers’ own health and well-being and self-management skills in caregiving, which can be termed emotional/informational support. These may explain the significant association between J-IEXPAC CAREGIVERS and the family caregivers’ participation in health checkups.

Focusing on social support among family caregivers, as Messina and Lane et al., noted, peer group participation is a key element in improving social support in cases in which existing social network members do not provide the necessary support. The introduction of a new network of individuals experiencing similar stressors enhances emotional/informational support and may promote preventive health behaviors [16]. J-IEXPAC CAREGIVERS includes an item to assess whether professionals encourage family caregivers to participate in peer groups. Thus, family caregivers with high J-IEXPAC CAREGIVERS scores may be encouraged to participate in informal peer groups with formal support from professionals, thereby enhancing their emotional/informational support and contributing to the promotion of preventive health behaviors, i.e., their participation in health checkups.

Although these possible mechanisms account for the attention for the caregiver domain in J-IEXPAC CAREGIVERS, it is interesting that the results of this study also show a significant association between attention for the patient and caregivers’ participation in health checkups. This may be due to the strong relationship between the two domain scores [24]. Caregivers may devote years of their own lives to caring for a loved one with chronic illness, and the needs and interdependence of the patient and family increase as the family ages. Professionals are required to provide comprehensive care for patients and their family caregivers [37]. In this study, interprofessional care for both patient and family caregiver (not just for the patient or the family caregiver) and the caregiver’s perception of this, may have contributed to the caregiver’s preventive health behavior.

To our knowledge, this study is the first to reveal an association between family caregivers’ experience of interprofessional care and their preventive health behaviors. The IEXPAC CAREGIVERS is an established measure for the evaluation of family caregivers’ experience of health and social care in Spain and Japan, and represents the quality of integrated care for both patient and caregiver. The findings of this study may underscore the clinical significance of evaluating the quality of care in terms of the family caregivers’ experience. Family caregivers may be able to stay healthy and continue to care longer [4] when they feel that professionals provide integrated health and social care.

Our study has several potential limitations. First, there are some potential unmeasured confounders: self-rated health, which is often used as a confounder in testing preventive health behaviors; informal social support variables, including the presence of multiple family caregivers; working; routine visit to hospitals and/or clinics; patient experience (the quality of primary care that caregivers experience as patients), which has been demonstrated to be linked to clinical process of preventive care [38,39]. Before a variable can be considered a potential confounder, it must be shown to be associated with the exposure [40]. Similarly, to determine whether these present variables can be considered confounders, we need to examine associations between them and J-IEXPAC CAREGIVES in future studies. Second, the study participants may not be representative of Japanese family caregivers. Family caregivers who were interested in evaluating the quality of care might have been more likely to complete the questionnaires. Third, the data were cross-sectional, and a causal relationship between family caregiver experience of interprofessional care and participation in health checkups cannot be definitively established.

## 5. Conclusions

We found that better family caregiver experience of interprofessional care for patients and caregivers was associated with caregiver participation in health checkups. These results support the significance of family caregiver experience of care, which may promote preventive health behaviors, such as participation in health checkups.

## Figures and Tables

**Table 1:** Participant characteristics by caregiver participation in health checkups in the past year.

Characteristic	Total		Participation in Health Checkups				
	(*n* = 251)		Yes (*n* = 182)		No (*n* = 69)		*p* Value
**Caregivers**							
Gender							
Men	56	(22.3%)	41	(22.5%)	15	(21.7%)	0.893
Women	195	(77.7%)	141	(77.5%)	54	(78.3%)	
Data missing	0						
Education							
Less than high school	21	(8.4%)	10	(5.5%)	11	(15.9%)	0.019
High school	126	(50.2%)	90	(50.0%)	36	(52.2%)	
Junior college or vocational college	61	(24.3%)	46	(25.6%)	15	(21.7%)	
College or graduate school	40	(15.9%)	34	(18.9%)	6	(8.7%)	
Data missing	3						
Equivalent income (million Japanese yen)							
Q1 (<1.06)	62	(24.7%)	30	(16.5%)	32	(46.4%)	<0.001
Q2 (1.06–2.01)	59	(23.5%)	43	(23.6%)	16	(23.2%)	
Q3 (2.02–3.16)	56	(22.3%)	41	(22.5%)	15	(21.7%)	
Q4 (≥3.17)	66	(26.3%)	61	(33.5%)	5	(7.2%)	
Data missing	8						
**Patients**							
Care-need level							
Lower (support required 1 and 2)	36	(14.3%)	25	(13.7%)	11	(15.9%)	0.277
Middle (care required 1–3)	155	(61.8%)	118	(64.8%)	37	(53.6%)	
Higher (care required 4 and 5)	55	(21.9%)	36	(19.8%)	19	(27.5%)	
Data missing	5						
**Caregivers**	Median (IQR)						
Age (years, *n* = 251)	64	(58–70)	63	(58–69)	66	(57–72.5)	0.078
Caregiver burden score (points, *n* = 247)	11	(6–17)	11	(6–16)	10.5	(5–18)	0.834

Q, Quartile. IQR, interquartile range. Comparisons of proportions were made using chi-square test. Comparisons of medians were made using the Mann–Whitney test.

**Table 2:** Distribution ^1^ of J-IEXPAC CAREGIVERS and unadjusted associations with caregiver participation in health checkups.

		Family Caregiver Participation in Health Checkups		95% CI	*p*
	Total (*n* = 234)	Yes (*n* = 170)	No (*n* = 64)		
J-IEXPAC CAREGIVERS Total score ^2^	40.5 (8.8)	41.3 (8.5)	38.4 (9.1)	0.4–5.4	0.025
	Total (*n* = 241)	Yes (*n* = 175)	No (*n* = 66)		
Domain score Attention for the patient ^3^	25.6 (5.3)	26.1 (5.1)	24.3 (5.6)	0.3–3.3	0.019
	Total (*n* = 247)	Yes (*n* = 179)	No (*n* = 68)		
Domain score Attention for the caregiver ^4^	12.9 (3.3)	13.2 (3.3)	12.2 (3.3)	0.1–2.0	0.032

^1^ Mean (SD). ^2^ Possible score lies in the range 12–60. ^3^ Possible score lies in the range 7–35. ^4^ possible score lies in the range 4–20. Independent samples *t*-test.

**Table 3:** Associations of the J-IEXPAC CAREGIVERS total score and other variables with caregiver participation in health checkups (*n* = 251).

	OR	(95% CI)	*p* Value
**J-IEXPAC CAREGIVERS total score ^1^**	1.05	(1.01–1.09)	0.013
Gender			
Men	Reference		
Women	1.07	(0.49–2.35)	0.864
Age (years)	1.01	(0.97–1.05)	0.683
Education			
Less than high school	Reference		
High school	1.63	(0.58–4.57)	0.357
Junior college or vocational college	1.19	(0.35–4.04)	0.785
College or graduate school	2.35	(0.60–9.87)	0.244
Equivalent income (million Japanese yen)			
Q1 (<1.06)	Reference		
Q2 (1.06–2.01)	2.54	(1.12–5.74)	0.026
Q3 (2.02–3.16)	2.76	(1.21–6.32)	0.016
Q4 (≥3.17)	15.21	(4.71–49.15)	<0.001
Caregiver burden score ^1^	0.99	(0.95–1.04)	0.732
Care-need level			
Lower (support required 1 and 2)	Reference		
Middle (care required 1–3)	1.41	(0.57–3.51)	0.457
Higher (care required 4 and 5)	0.59	(0.21–1.66)	0.317

^1^ per 1-point increase. OR, odds ratio. Q, Quartile.

**Table 4:** Associations of two domain scores of J-IEXPAC CAREGIVERS and other variables with caregiver participation in health checkups (*n* = 251).

	OR	(95% CI)	*p* Value
**Attention for the patient ^1^**	1.08	(1.01–1.15)	0.016
Gender			
Men	Reference		
Women	1.07	(0.49–2.33)	0.874
Age (years)	1.01	(0.97–1.05)	0.662
Education			
Less than high school	Reference		
High school	1.58	(0.56–4.45)	0.386
Junior college or vocational college	1.13	(0.33–3.84)	0.847
College or graduate school	2.10	(0.50–8.77)	0.313
Equivalent income (million Japanese yen)			
Q1 (<1.06)	Reference		
Q2 (1.06–2.01)	2.61	(1.15–5.92)	0.026
Q3 (2.02–3.16)	2.91	(1.27–6.64)	0.016
Q4 (≥3.17)	15.50	(4.79–49.99)	<0.001
Caregiver burden score ^1^	0.99	(0.95–1.04)	0.723
Care-need level			
Lower (support required 1 and 2)	Reference		
Middle (care required 1–3)	1.40	(0.56–3.46)	0.470
Higher (care required 4 and 5)	0.58	(0.21–1.64)	0.307
**Attention for the caregiver ^1^**	1.12	(1.02–1.24)	0.023
Gender			
Men	Reference		
Women	1.10	(0.50–2.40)	0.819
Age (years)	1.01	(0.97–1.05)	0.708
Education			
Less than high school	Reference		
High school	1.68	(0.60–4.73)	0.323
Junior college or vocational college	1.20	(0.35–4.11)	0.768
College or graduate school	2.60	(0.61–10.98)	0.195
Equivalent income (million Japanese yen)			
Q1 (<1.06)	Reference		
Q2 (1.06–2.01)	2.51	(1.11–5.67)	0.026
Q3 (2.02–3.16)	2.64	(1.15–6.06)	0.016
Q4 (≥3.17)	15.01	(4.66–48.36)	<0.001
Caregiver burden score^1^	0.99	(0.95–1.04)	0.731
Care-need level			
Lower (support required 1 and 2)	Reference		
Middle (care required 1–3)	1.39	(0.56–3.45)	0.473
Higher (care required 4 and 5)	0.61	(0.22–1.70)	0.344

^1^ per 1-point increase. OR, odds ratio. Q, Quartile.

## Data Availability

The data presented in this study are available on request from the corresponding author. The data are not publicly available due to ethical considerations.
